# Hatchery efficiency for turtle conservation in Cabo Verde

**DOI:** 10.1016/j.mex.2021.101518

**Published:** 2021-09-16

**Authors:** Samir Martins, Nádia Ferreira-Veiga, Zuleika Rodrigues, Adélcio Querido, Nuno de Santos Loureiro, Kátia Freire, Elena Abella, Carolina Oujo, Adolfo Marco

**Affiliations:** a Faculty of Sciences and Technology, University of Algarve, Gambelas, P-8005-139 Faro, Portugal; b BIOS.CV - Conservation of the Environment and Sustainable Development, CP 52111, Sal Rei – Boa Vista Island, Cabo Verde; c Estación Biológica de Doñana, CSIC, C/ Américo Vespucio, s/n, 41092 Sevilla, Spain

**Keywords:** Loggerhead turtle, Egg mortality, Clutch relocation, Conservation, Cabo Verde

## Abstract

This paper evaluated the efficiency of beach hatcheries as a conservation tool for threatened sea turtle clutches. During six nesting seasons (2013 to 2018), several thousand high-risk clutches from loggerhead turtles (Caretta caretta) were relocated to a hatchery constructed on the same beach, within the Sea Turtle Natural Reserve (STNR, Boa Vista Island, Cabo Verde). Some parameters like hatching success; incubation period, hatchlings’ morphology and their behavioral response were compared to in-situ clutches.

• Our findings confirmed that the in-situ nests within the STNR had extremely high egg mortality that was usually over 70 %. Mean hatching success of clutches relocated to hatcheries was significantly higher than in-situ clutches with mean values between 70 to 85 % (*p* < 0.0001).
• No significant differences were observed in the incubation period (*p* = 0.786) and morphology of hatchlings (all *p* > 0.05) between relocated and in-situ clutches.
• This study provided a detailed method and recommendations for sea turtle clutches relocation to the hatchery, that can be beneficial for endangered sea turtle population specially where hatching success is very low.

## Graphical abstract

**Figure:**
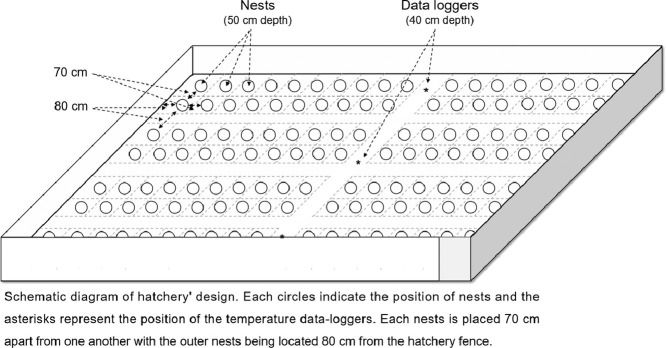

Specifications table

**Table:** 

Subject Area:	Environmental ScienceEnvironmental Science
More specific subject area:	*Relocation of doomed sea turtle clutches to hatchery*
Method name:	*Clutches relocation*
Name and reference of original method:	*Hatchery efficiency as a conservation tool in threatened sea turtle rookeries with high embryonic mortality.* *The manuscript is accepted for publication in Ocean and Coastal Management (Article reference: OCMA_105807).*
Resource availability:	*None*

## Method details

In this study, we evaluate the potential impact of clutch relocation on hatching output. Moreover, we evaluate the impact of the relocation process and the controlled incubation in hatcheries on the phenotype and viability of the offspring, comparing hatchlings from in-situ and relocated clutches. In the study area, Boa Vista Island, Cabo Verde, multiple clutch relocation programs to beach hatcheries consisting of several hundred clutches per season are implemented to maximize hatchling survival due the high mortality by tidal flooding and ghost crab predation [8], [9], [10], [11], [12].

### Beach patrols

The beach was monitored nightly during six nesting seasons (2013-2018) to find all clutches laid by the loggerhead turtles. During the beach patrol, standard protocols (Schroeder & Murphy, [7]) were followed.

Nests were marked by experienced staff members during the patrols from mid-June until the end of September. The number of eggs was counted after the turtle began camouflaging. To reduce human influence, the female was not approached until she had finished camouflaging the nest.

To compare the hatchlings’ morphologies between in-situ and hatchery clutches, during the 2015 nesting season, 64 additional nests were protected with a circular plastic mesh to trap hatchlings to enable them to be counted and sampled after emergence.

The nests were monitored every day before sunrise, recording all information of occurrences that could potentially affect the nest, such as ghost crab predation or tidal flooding. Nests were excavated after 60 days or after a large group emergence. The emergence was identified by the tracks of hatchlings in the sand.

### Nest Relocation

In the beginning of the nesting season, a hatchery (16 × 27 m; N16.016 W22.741) was built in João Barrosa beach in the same location for each of the different study seasons (2013–2018), in an area easily accessible from the field camp with less than 10 min walking distance. To prevent tidal flooding, an area of higher ground was chosen and the hatchery was constructed in a manner that would prevent ghost crabs and nesting females from entering.

Clutches were carefully relocated to the hatchery after they were laid. During 2013 to 2018 the same clutch relocation method was used following tested standard protocols [2]. The clutches were reburied in standardized hand-dug cavities that resembled in-situ nests in terms of shape, size and sand characteristics. The hole was dug using a ruler to ensure every nest was dug at the same depth, 50 cm which represents the average depth for this population [8]. In the first 30 cm, the hole was 25 cm wide, bellow the depth of 30 cm, a circular chamber was made 35 to 40 cm wide. The sand used to build the hatchery was taken from the same beach area. The priority was to relocate clutches which had been laid in an area with high risk of flooding and also in vegetated or muddy areas. We used the same protocols during all study seasons (2013–2018).

Each clutch was placed 70 cm apart from one another with the outer nests being located 80 cm from the hatchery fence. All nests were marked with sticks that were numbered and dated. A circular plastic mesh was positioned on the surface above each nest, trapping the hatchlings when they emerged to enable them to be counted and sampled. During this period, nests were monitored from dusk until dawn. The total time between nesting and reburying never surpassed 6 hours. Nests were excavated at least 4 days after the last emergence event or after more than 60 days of incubation.

### Hatchlings and nest excavations

Twenty hatchlings were randomly selected from 39 nests on the beach and 160 nests in the hatchery to measure their straight carapace length (SCL) and straight carapace width (SCW) using a digital calliper (Digital Vernier Calliper 150 mm/6 inch; ± 0.1 mm) and their body mass was weighed with an electronic scale (Mission Quark Pocket Scales - Weigh Darts; ± 0.1 g). Afterwards, a righting response test was conducted once for each hatchling. For the righting response test, the hatchlings were turned over, placed on a smooth sand surface and allowed to turn by themselves while the time was recorded [12,14]. Each hatchling was tested during a maximum time of 60 s. For hatchlings that did not exhibit a righting response after the limit of 60 s, that time was assigned to them. Handling time did not exceed 15 min with the hatchlings being immediately released after sampling.

### Sand temperature

During each nesting season, temperature data loggers (*n* = 3, Stow Away TidbiT® HOBO with accuracy of ± 0.2 °C) were buried in a line along the center of the hatchery 3 m from each other (Supplementary Fig. S1). They were positioned >1 m away from any clutches and at an average depth of 40 cm to record the sand temperature. Additionally, three data loggers were buried in the sand in different areas along the length of the beach (one at either end and one in the centre) at the same depth (40 cm). This corresponds to the mean depth at which the middle of a clutch of eggs is found for this population of loggerheads [2,8]. All dataloggers were calibrated before being buried in the sand. They were programmed to take one measurement every 30 min, from the beginning of July to mid-November. The temperature record allows us to determine if the temperature in the hatchery is too hot or too cool in relation to the beach sand temperature.

## Method validation

Our results indicated that in our study area, loggerhead clutches suffered high mortality in in-situ nests by ghost crabs and flooding and relocating the doomed eggs to a hatchery reduced these risks. No significant differences were observed in the incubation period (*p* = 0.786) and hatchlings morphologies (all *p* > 0.05) among in-situ and hatchery clutches. We suggest that implementing a hatchery is done based on a case-by-case evaluation as the pressures on a population may differ from one region to another. However, we do not recommend the use of hatcheries without careful consideration of local ecological conditions and conservation priorities.

For better hatchery management, a series of guidelines have been suggested. The first one is the site selection [13] which should preferentially be on the same beach and located at sufficient elevation to prevent damage from flood intrusion and at a safe distance from any plant roots [1,13]. Hatchery fence should be built using wood and plastic material and metal mesh should be avoided. For instance, a study suggested that hatchlings use earth's magnetic field for migration, therefore, the metal material may harm the local magnetic field and disorient the hatchlings [6]. The bottom of the fence should be 40–50 cm deep to prevent the access of predators like ghost crabs.

Previous training is required for all staff or volunteers who will participate in the relocation process in order to standardize the methodology [3]. Extreme care should be taken during egg collection, transport and handling. Throughout egg handling, a high standard of hygiene is necessary and the use of chemicals such as mosquito repellent on hands should be avoided. The relocation can occur at any time, however, sun exposure must be avoided [2]. Clutches that are more than six hours old need to be treated with extreme care. It should be guaranteed that they maintain their original vertical orientation and vibrations should be avoided [2]. In addition, the clutch should be counted and placed at the original nest depth or in the mean nest depth common in the rookery [8]. All the clutches should be fenced by plastic mesh to prevent hatchlings from escaping after the emergence and the hatchery fence should efficiently prevent predators from entering. In areas where human exploitation occurs, the hatchery must be guarded at all times.

It is important to accurately count the number of hatchlings that emerge in each nest to determine the emergence success [13]. During the nest exhumation, all the of the eggshells and unhatched eggs must be removed from the hatchery. The nest exhumation in the hatchery is essential to understanding the hatching success rate of the relocation program and compare it with the productivity of the nests on the beach. Which it is very important to help identify the cause of egg mortality in the hatchery, to reduce the mortality of relocated eggs and improve the effectiveness of the threatened nest in the relocation program.

In cases of rookeries that are vulnerable to high environmental temperatures that lack coastal vegetation, it is recommended to create a shade for the nest or water the sand in the hatchery to mitigate the negative impacts of climate change on the clutch's sex ratio and hatchling's fitness [4], [5]. On the other hand, in the cases of hatcheries with cooler temperatures than the beach it is suggested to deposit a layer of black sand, 2 to 3 cm thick, over the surface of each nest area, immediately after the clutch relocation [12].

## Appendix Supplementary materials

Image, application 1
