# Supplementary material for: Hatchery efficiency for turtle conservation in Cabo Verde
Source: MethodsX. 2021 Sep 16;8:101518. doi: 10.1016/j.mex.2021.101518 (PMC8563676; doi:10.1016/j.mex.2021.101518)
Supplement: Supplementary file 1 [file mmc1.docx]

Appendix A. Supplementary data

Figure S1. Aerial photograph of João Barrosa beach, Boa Vista Island (A) with the hatchery (white rectangle). Zoom of hatchery zone, showing the position of dataloggers into the hatchery. Orthophoto of Boa Vista — Cape Verde (icloud.com).


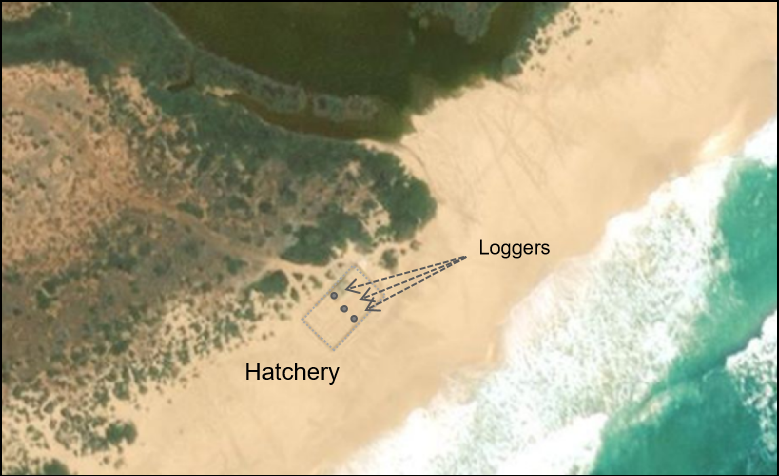

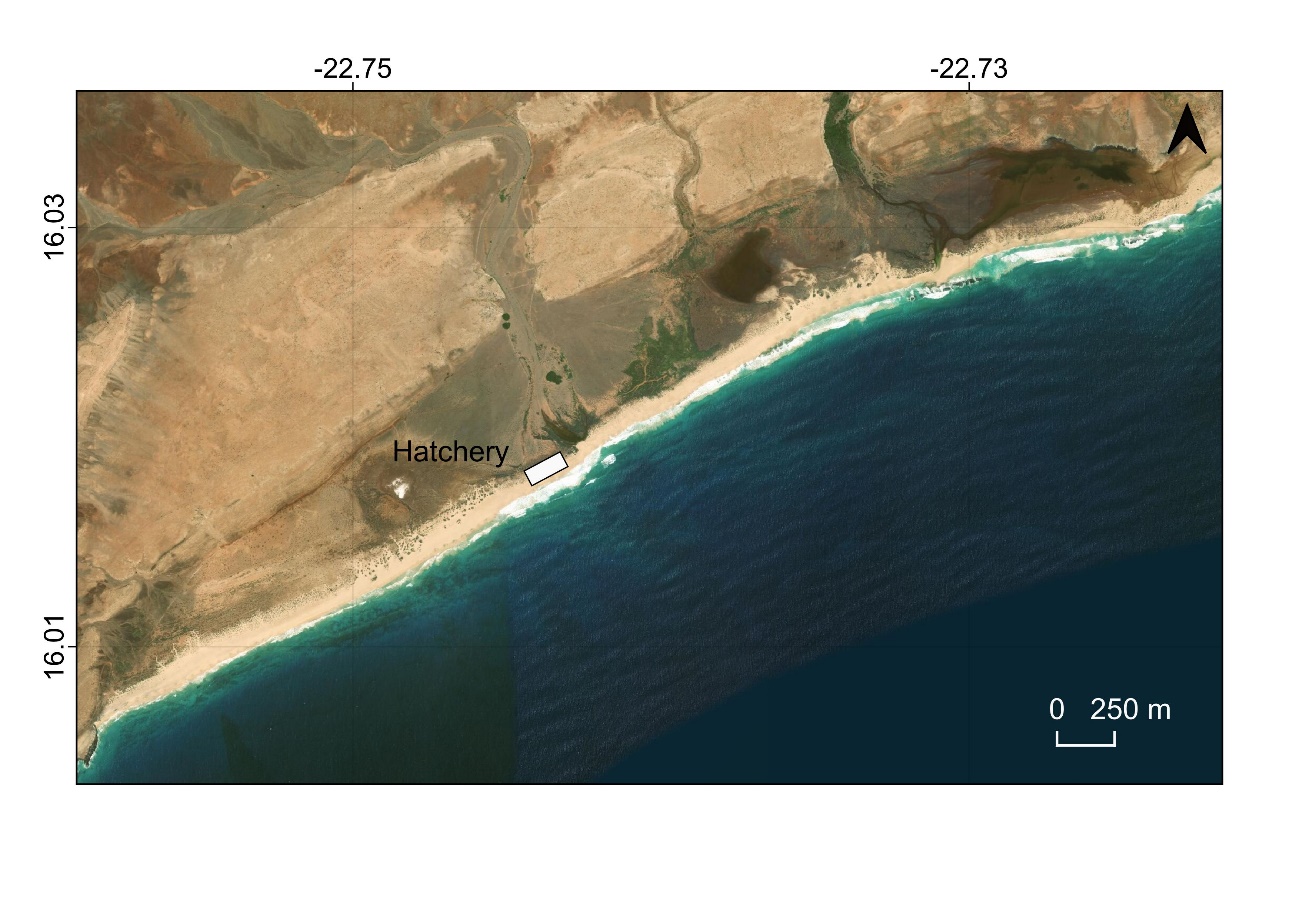


**A**

**B**
